# Molecular Pathogenesis and Treatment Perspectives for Hypereosinophilia and Hypereosinophilic Syndromes

**DOI:** 10.3390/ijms22020486

**Published:** 2021-01-06

**Authors:** Stefania Stella, Michele Massimino, Livia Manzella, Maria Stella Pennisi, Elena Tirrò, Chiara Romano, Silvia Rita Vitale, Adriana Puma, Cristina Tomarchio, Sandra Di Gregorio, Giuseppe Alberto Palumbo, Paolo Vigneri

**Affiliations:** 1 Department of Clinical and Experimental Medicine, University of Catania, 95123 Catania, Italy; michedot@yahoo.it (M.M.); manzella@unict.it (L.M.); perny76@gmail.com (M.S.P.); ele_tir@yahoo.it (E.T.); chiararomano83@gmail.com (C.R.); silviarita.vitale@gmail.com (S.R.V.); adry.p88@hotmail.it (A.P.); cristina.tomarchio@hotmail.it (C.T.); digregoriosandra@hotmail.com (S.D.G.); vigneripaolo@gmail.com (P.V.); 2 Center of Experimental Oncology and Hematology, A.O.U. Policlinico “G.Rodolico-San Marco”, 95123 Catania, Italy; 3 Department of Scienze Mediche Chirurgiche e Tecnologie Avanzate “G.F. Ingrassia”, University of Catania, 95123 Catania, Italy; giuseppealberto.palumbo@gmail.com

**Keywords:** hypereosinophilia, hypereosinophilic syndromes, PDGFRα and PDGFRβ fusions, NGS, TCR rearrangements

## Abstract

Hypereosinophilia (HE) is a heterogeneous condition with a persistent elevated eosinophil count of >350/mm^3^, which is reported in various (inflammatory, allergic, infectious, or neoplastic) diseases with distinct pathophysiological pathways. HE may be associated with tissue or organ damage and, in this case, the disorder is classified as hypereosinophilic syndrome (HES). Different studies have allowed for the discovery of two major pathogenetic variants known as myeloid or lymphocytic HES. With the advent of molecular genetic analyses, such as T-cell receptor gene rearrangement assays and Next Generation Sequencing, it is possible to better characterize these syndromes and establish which patients will benefit from pharmacological targeted therapy. In this review, we highlight the molecular alterations that are involved in the pathogenesis of eosinophil disorders and revise possible therapeutic approaches, either implemented in clinical practice or currently under investigation in clinical trials.

## 1. Introduction

### 1.1. Eosinophil Development

Eosinophils are white blood cells of the granulocytic lineage that play an important role in innate immune functions [1] and develop in bone marrow from pluripotent stem cells expressing CD34^+^CD125^+^ antigens. These cells represent about 5% of the circulating blood leukocytes with an absolute eosinophil count (AEC) in healthy adults that is usually between 350 and 500/mm^3^, which increases during inflammatory processes, such as allergic diseases, parasitic, bacteria, and virus infection [2,3].

Structurally, they possess segmented bi-lobed nuclei and specific primary and secondary granules. Primary granules exhibit lysophospholipase activity that is involved in eosinophilic-dependent tissues inflammation [4]. Secondary granules contain many mediators, such as major basic protein (MBP), eosinophil cationic protein (ECP), eosinophil peroxidase (EPO), and eosinophil-derived neurotoxin (EDN), which are all able to induce both inflammation and tissue damage [5].

Furthermore, eosinophils are equipped of lipid bodies that play a critical role in asthma, as they cause eicosanoidis production [6]. Finally, they are potent productors of both reactive oxygen species and nitric oxide, which promote the anti-bacterial activity, while the ability to internalize the respiratory syncytial (RSV) and influenza viruses document the role of eosinophils in the viral response [7,8].

### 1.2. Eosinophil Contents, Biology and Homeostatic Immune Role

Detecting tissue-resident eosinophils showed that they are distributed in heart, skin, lung, and kidneys [9]. Despite this observation, under the homeostasis condition, eosinophils are particularly abundant in the gastrointestinal tract (GI), where they are involved in different biological processes. Both a beneficial and nonbeneficial role of eosinophils in GI tract have been postulated.

The first role is based on the ability of gastrointestinal eosinophils to mediate anti-parasitic response and promote with them a symbiotic association that is aimed at the maintenance of tissue homeostasis. Moreover, in the GI tract, a high number of eosinophils trapping bacteria represents an effective mechanism to protect this tissue from bacterial invasion.

The nonbeneficial role of gastrointestinal eosinophils is reported in Eosinophil Gastrointestinal Disorders (EGIDs) and Inflammation Bowel Disease (IBD). The pathogenesis of both diseases is dependent on tissue infiltration of eosinophils, followed by the accumulation of activated immune cells, such as B and T cells, as well as the production of pro-inflammatory cytokines [8].

An additional particular function of eosinophil is their role in tissue regeneration and remodeling. By the secretion of IL-4, eosinophils are able to facilitate liver and muscle regeneration [10,11], while the increased presence of these cells in the endometrium prompted speculation that they may play a role in tissue remodeling during ovulation and menstruation [12]. The absolute number and biology of eosinophils are both usually controlled by type-2 cytokines, such as interleukin-5 (IL-5), granulocyte-macrophage colony-stimulating factor (GM-CSF), and interleukin-3 (IL-3), produced by T-lymphocytes, mast cells, and stromal cells. IL-5, IL-3, and GM-CSF induce eosinophils maturation, survival, and apoptosis inhibition by PI3K, ERK, and STATs pathway activation. However, in this biological system, IL-5 shows a more prominent role than IL-3 and GM-CSF dependent on its high specificity for this leukocyte subset. In particular, the IL-5 receptor (IL-5R) plays a central role of intracellular signals regulation by its α and β-chains. The α-chain contains the ligand-binding subunit while the β-chain is defined as non-ligand-binding subunit and it mediates the intracellular signal transduction. The β-chain, in turn, is shared with IL-3 and GM-CSF receptors (IL-3R) (GM-CSFR), thus supporting the intriguing role of IL-5 as central mediator of type-2 cytokines-dependent eosinophil survival. An interestingly involvement of IL-5 concerns its production by group 2 innate lymphoid cells (ILC2s) after interleukin-33 (IL-33) stimulation. IL-33 promotes the IL-5 production from ILC2s, which, in turn, releasing IL-5 improves eosinophils expansion and survival. Hence, ILC2s are a new important mediator of IL-33-driven eosinophil disorder development [13,14].

Hence, IL-5, to date, is an attractive therapeutic target for the treatment of eosinophil-mediated disorders [15]. Nevertheless, despite these results, the published data report that IL-5 over-expression or down-regulation, alone, fail to induce eosinophil-mediated damage or eosinophils maturation [16], making its role unclear. However, all together, these observations implicate that the deregulation of IL-3, IL-5, and GM-CSF signaling may cause HES [15,17,18].

Finally, an important role in eosinophils biology concerns the fibrogenic activity of transforming growth factor beta-1 (TGF-B1). Eosinophils are a strong productor of TGF-B1, which is involved in airway remodeling or in disease state in different tissue, such as skin (atopy), nose (nasal polyposis), and blood (idiopathic hypereosinophilic syndrome) [19].

Eosinophils biology is also regulated by the cell surface glycan-binding protein, named siglecs (sialic acid immunoglobulin-like lectins). The most important glycan in eosinophils biology is Siglec-8 (originally named sialoadhesin family 2—SAF-2), as it is expressed selectively in these leukocyte types. Siglec-8 is stimulated by sialic acids, which induce the activation of two tyrosine-based motifs, defined immunoreceptor tyrosine-based inhibitor motif (ITIM) and a membrane-distal immunoreceptor tyrosine-based switch motif (ITSM), thus initiating the downstream receptor functions. Siglec-8 is involved in reactive oxygen species (ROS) production, in the loss of mitochondrial membrane potential, ERK1/2 activation, and caspase cleavage modulating apoptosis and cell survival [20]. For these regions, Siglec-8 was studied as a therapeutic target in patients with eosinophilic disorders using chimeric antibodies [21].

### 1.3. Eosinophil Recruitment into Blood and Tissue, Survival and Death

The term eosinophilia is employed for a small increase of the AEC in the blood (up to 1500/mm^3^), while hypereosinophilia (HE) indicates an AEC greater than 1500/mm^3^ on two consecutive blood samples drawn at a one-month interval. This persistent eosinophilia is usually linked to helminth infections, allergies, atopy, drugs, neoplastic disorders, or autoimmune diseases [22,23].

A second type of hypereosinophilia is tissue HE, which is defined as a percentage of eosinophils in the bone marrow (BM) that exceeds 20% of all nucleated cells, followed by extensive tissue infiltration, such as skin (in 69% of subjects), lung, and the gastrointestinal tract (44% and 38%, respectively) [22,23,24].

Upon activation, eosinophils infiltrate tissues, degranulate, and release proinflammatory cytokines that cause organ damage and dysfunction, defined as the Hypereosinophilic Syndrome (HES). HES represents a group of heterogeneous disorders characterized by persistent and unexplained HE in the blood or peripheral tissues usually associated with multiple organ damage or dysfunction. This damage may be due to direct cytotoxic effects of the eosinophil granulate contents or may occur because of the secondary involvement of other cell types [24,25]. Once eosinophils leave the blood circulation and migrate into tissue sites, they do not recirculate. Into the tissue, the survival is dependent on local production of cytokines that also prevent eosinophil apoptosis for several days. In fact, the patterns of cytokines regulate the recruitment of eosinophils to the specific tissue sites, activate endothelial cells, and induce tissue-resident cells to produce eosinophil-active chemokines to facilitate their preferential migration [26].

The cardiovascular system is often involved in HES [27,28].

The presenting symptoms of HE and HES are variable and they may include weakness, fatigue, cough, dyspnea and rhinitis, myalgias or angioedema, rash or fever, as well as severe tissue damage or end-organ failure [23,29]. Leukocytosis, anemia, abnormal platelet counts, increased vitamin B12 (>1000 pg/mL), and tryptase (>12 ng/mL) levels represent additional alterations that are associated with the disease [23,24].

Because HES is a rare neoplasm, its epidemiology has not been accurately investigated. Hence, the disease true incidence is unknown, which is mainly due to the lack of specific coding for the different HES variants. Although HES is mainly diagnosed in adults, 20 to 50 years old, it can also affect children and the elderly [30].

The aim of this review is to outline the molecular alterations that are involved in the pathogenesis of eosinophil disorders and provide an update on the therapeutic approaches that are available for the treatment of these disorders.

## 2. Classification of Hypereosinophilia and Hypereosinophilic Syndrome

HE and HES can usually be divided into multiple subgroups based upon clinical, laboratory, and molecular features.

HE is classified in three groups: primary (neoplastic or clonal), secondary (reactive), and idiopathic [24]. Primary HE is caused by a clonal stem cell disorder affecting the myeloid or lymphoid lineage of the malignant clone, while secondary HE can be associated with both pathological phenomena (e.g., parasitic infections, allergies, autoimmune disorders) and/or non-myeloid or solid tumors, in which eosinophilia results from the production of cytokines by malignant cells, such as in peripheral T-cell lymphoma and classical Hodgkin lymphoma. If the etiology is not primary or secondary and the HE persists for six or more months, then the disease is defined as Idiopathic HE (HE_US_) [30]. Moreover, a rare familiar form of HE was recently identified that is characterized by autosomal dominant inheritance with a benign clinical course and seldom characterized by organ dysfunction [23,24,31].

Over the years, the Working Conference on Eosinophil Disorder and Syndromes proposed various terminologies for eosinophilic syndromes. The HE subtypes were then divided into a hereditary (familial) variant (HE_FA_), HE of undetermined significance (HE_US_), primary (clonal/neoplastic) HE produced by clonal/neoplastic eosinophils (HE_N_), and secondary (reactive) HE (HE_R_), with the latter group including the lymphocyte variant as a subtype. The HE_US_ acronym was introduced as a novel term instead of idiopathic HE [30].

Similar to the HE classification, HES can be grouped in primary (neoplastic), secondary (reactive), and idiopathic (undetermined significance), respectively, named HES_N_, HES_R_, and HES_US_ [23].

**Figure 1:**
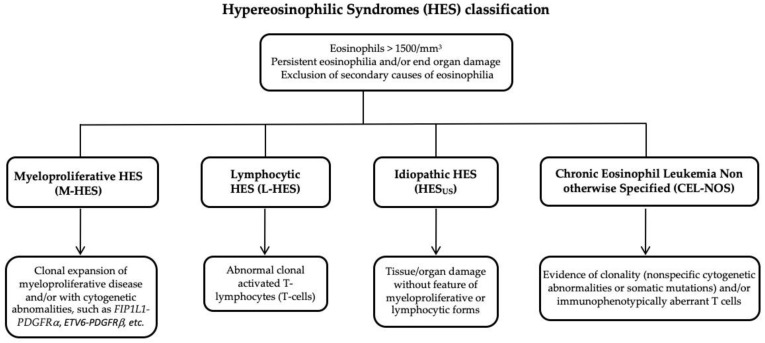
Hypereosinophilic syndromes (HES) classification.

The main two pathogenic forms of HES are the myeloproliferative (M-HES) and lymphocytic (L-HES) forms of the disease, respectively, classified as *Myeloid Hypereosinophilic Syndrome* and *Lymphocytic Hypereosinophilic Syndrome.* Each group includes several clinically distinct HES disorders [32]. Patients that do not display the M- or L-HES diagnosis can be classified as being affected by a *Idiopathic Hypereosinophilic Syndrome* or a *Chronic Eosinophilic Leukemia non otherwise specified* (CEL-NOS) (Figure 1).

### 2.1. Myeloid Hypereosinophilic Syndrome

Patients with M-HES are characterized by hepatomegaly, splenomegaly, circulating myeloid precursors, increased serum vitamin B_12_ and/or tryptase levels, anemia, thrombocytopenia, hematologic diseases (myeloid fibrosis, left shift in maturation of myeloid precursors), and/or cytogenetic abnormalities [27,31,33,34]. The primary molecular defect that is responsible for this distinct phenotype is a gene fusion between *FIP1-like* 1 (*FIPL1*) and platelet-derived growth factor receptor alpha (*PDGFRα*), known as *FIP1L1–PDGFRα* fusion. Several other fusions involve genes encoding for the *PDGFRα, PDGFRβ,* fibroblast growth factor receptor 1 (*FGFR1),* breakpoint cluster region *(BCR),* janus kinase 2 *(JAK2),* fms-like tyrosine kinase 3 *(FLT3),* and Abelson tyrosine kinase 1 *(ABL1)* genes. Recently, the WHO added the *periocentriolar material 1 (PCM1)-JAK2* fusion in the classification of this group [32,35]. Rarely, *PDGFRβ* rearrangements are cryptic, even if patients with this anomaly (involving over 30 gene fusion partners) can present a disease without eosinophilia [23]. M-HES related to gene fusions involving the *FGFR1* gene are rare, although several groups reported up to 14 different *FGFR1* gene partners [30,35].

In the last decade, cytogenetics and DNA sequencing have allowed for investigating the molecular alterations found in HES, demonstrating that somatic mutations are usually uncommon in patients harboring *PDGFRα, PDGFRβ*, or *PCM1-JAK2* rearrangements, but significantly more frequent in *FGFR1* rearranged cases [24].

### 2.2. Lymphocytic Hypereosinophilic Syndrome

The lymphocytic variant of HES is a less clearly defined disease entity that is characterized by the overproduction of eosinophilopoietic cytokines (IL-5 and/or IL-3) causing the recruitment of clonal activated T-lymphocytes (T-cells). IL-5 overproduction by T-cells is responsible for blood and tissue HE, which leads to clinical manifestations, while the expansion of the abnormal T-cell subset is usually asymptomatic, with the exception of a few cases that evolve to T-cell lymphoma [27]. Immunophenotypically, these abnormal T cells include double-negative cells, immature T-cells (e.g., CD3^+^CD4^−^CD8^−^), or cells without CD3 expression (e.g., CD3^−^CD4^+^). Furthermore, additional immunophenotypic abnormalities include high CD5 expression on CD3^−^CD4^+^ cells and the loss of the CD7 surface marker and/or expression of CD27 [23,36,37].

L-HES is found in 17–27% of subjects with unexplained eosinophilia or HES. Primary disease manifestations are superficial adenopathy (62%) with rheumatologic (29%), gastrointestinal (24%), pulmonary (19%), neurologic (10%), and cardiovascular (5%) organ involvement, but no significant lymphocytosis [27].

Moreover, elevated IgE and thymus and activation-regulated chemokine (TARC) in serum are common in patients with L-HES. Particularly, the detection of serum TARC levels, in addition to the increased production of cytokines, may provide additional support for a correct diagnosis [23,37,38].

### 2.3. Idiopathic Hypereosinophilic Syndrome and Chronic Eosinophilic Leukemia non Otherwise Specified

Idiopathic HES was defined as persistent HES with tissue/organ damage of unknown cause, whereas CEL-NOS presents clonal cytogenetic or molecular genetic abnormalities. In both syndromes, no rearrangements of *PDGFRα, PDGFRβ, FGFR1* or *PCM1-JAK2, ETS Variant Transcription Factor 1 (ETV6)-JAK2*, and *BCR-JAK2* fusion genes are present. In these cases, the cytogenetic and molecular alterations of chronic myeloid leukemia (CML), myelodysplastic/myeloproliferative neoplasms (MDS/MPN), chronic neutrophilic leukemia (CNL), and chronic myelomonocytic leukemia (CMML) should be excluded [39,40,41,42,43,44].

## 3. Molecular Pathogenesis in Hypereosinophilic Syndrome

The laboratory screening performed to formulate a HES diagnosis allow for us to understand molecular events that cause gene driver alterations in myeloid and lymphoid disorders that are associated with eosinophilia (summarized in Table 1).

**Figure 2:**
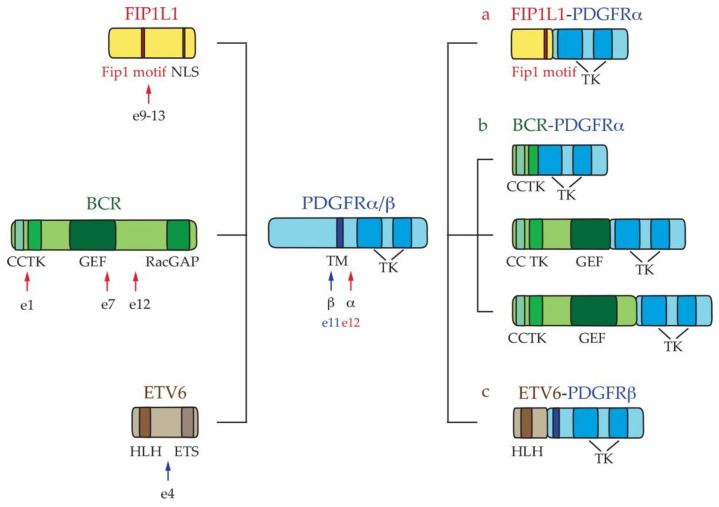
**Schematic representation of *platelet-derived growth factor receptor alpha* (*PDGFRα*) and *PDGFRβ* fusion rearrangements.** (a) *FIP1L1-PDGFRα*; (b) breakpoint cluster region-*PDGFR*α (*BCR-PDGFRα*); and, (c) *ETV6*-*PDGFRβ*. The arrows indicate the position of breakpoints of PDGFRs. BCR = breakpoint cluster region; CC = Coiled-Coil; ETS = (Erythroblast Transformation Specific) DNA-binding domain; ETV = ETS Variant Transcription Factor 1; FIP1L1 = Fip1-like1; GEF = Guanine Nucleotide Exchange Factor; HLH = Helix-Loop-Helix oligomerization domain; NLS = Nuclear Localization Signal; PDGFR = Platelet-Derived Growth Factor Receptor; RacGAP = COOH-terminal GTPase Activating Protein (GAP) domain; TM = Transmembrane; TK = Tyrosine Kinase.

*PDGFRα and PDGFRβ fusions*: *PDGFRα* and *PDGFRβ* are a class of receptors with TK activity, which are characterized by an extracellular ligand-binding region and two intracellular TK domains [45,46]. PDGFRα and β are monomeric transmembrane proteins that dimerize after binding PDGF, leading to TK domain activation. The activated catalytic domain promotes a cascade of signaling events via downstream pro-survival and anti-apoptotic effectors, such as SRC, STAT5, and the PI3K/RAS/MAP kinase pathway [47,48,49,50]. The most common *PDGFR* gene alterations reported in the eosinophilic syndrome are rearrangements with several partner genes, such as *FIP1L1, BCR*, and *ETV6* (Figure 2) [34].

*FIP1L1-PDGFRα fusions:* the *FIP1L1-PDGFRα* rearrangement represents the most frequently recurrent aberration in eosinophilia detected in different hematopoietic cells, including eosinophils, neutrophils, T-, or B-cells [51]. Although it is generally expressed in chronic myeloid neoplasms that are associated with eosinophilia, patients with lymphoblastic leukemia/lymphoma (T-ALL/LBL) or, less frequently, B-cell acute leukemia display this fusion transcript [23]. The *FIP1L1-PDGFRα* fusion protein is expressed in 10–20% of patients that are affected by HE_N_/HES_N_, with a higher prevalence in males [24].

The *FIP1L1-PDGFRα* transcript is generated by juxtaposition of the 5′ and 3′ regions of *FIP1L1* and *PDGFRα*, respectively. The fusion between these two genes is caused by an internal cryptic deletion, which is not detectable by cytogenetic banding techniques, resulting in an apparently normal karyotype. This deletion disrupts the juxtamembrane PDGFR*α* negative regulator domain, thus deregulating its TK activity [52,53,54,55]. The 5′-3′ juxtaposition involves exons 9–13 of *FIP1L1* and exon 12 of *PDGFRα*, which can generate different *FIP1L1-PDGFRα* splicing isoforms (not shown) (Figure 2a). In addition, an insertion of an additional *FIP1L1* intron sequence was identified in different patients [52,55,56].

Different researchers conducted several studies in human cell and in clonal HES mouse model in order to investigate the role of *FIP1L1-PDGFRα* in the pathogenesis of the disease. Several data were reported and, although the constitutive *FIP1L1-PDGFRα* kinase activation constrains a murine eosinophil-lineage commitment, in human hematopoietic progenitor cells promotes cytokine-independent colony formation without favoring eosinophil lineage by STAT5 and nuclear factor kappa-light-chain-enhancer of activated B cells (NFkB) activation [57,58,59]. In contrast, on the contrary of native *PDGFRα*, *FIP1L1-PDGFRα* does not activate the MAPK pathway, which suggests that its transforming properties do not require extracellular signal-regulated protein kinases 1 and 2 (ERK1/2) [49,52,60]. This effect is dependent on different subcellular localization of native or fusion proteins. Hence, while FIP1L1- PDGFRα has cytosolic location, PDGFRα is a transmembrane receptor that may access RAS, an upstream mediator of MAPK signaling [56]. However, other authors showed that FIP1L1- PDGFRα may activate the eosinophilic linage-specific transcription factors through RAS/MEK/p38 cascade [61].

*BCR-PDGFRα fusions:* several authors reported that, unlike the common BCR-ABL1 chimeric gene found in CML [62,63], the *BCR-PDGFRα* fusion detected in HES associates with different hematological diseases, such as atypical CML and pre-B ALL [64,65,66]. Molecular analysis revealed that the rearrangement process causes an in-frame mRNA fusion between exons 1, 7, or 12 of *BCR* and 12 or 13 of *PDGFRα* (Figure 2b). This molecular event disrupts the negative regulator domain of *PDGFRα* and catalyzes aberrant TK activity that is mediated by BCR-dependent oligomerization [56].

*ETV6-PDGFRβ fusions:* over 32 fusion genes involving the *PDGFRβ* have been discovered and *ETV6-PDGFRβ* represents the most frequent translocation t(5;12)(q33;p13) [30,35] found in eosinophilia associated with CML [67]. In the most common variant, the *ETV6-PDGFRβ* fusion transcript is generated by exons 4 and 11, respectively (Figure 2c) [68].

The extracellular *PDGFRβ* region contains the ligand binding site that is replaced by ETV6, which promotes an oligomerization process resulting in the activation of the *PDGFRβ* tyrosine kinase [69]. Studies that were conducted in murine models indicate that *ETV6*-*PDGFRβ* causes growth factor-independent proliferation of Ba/F3 cells and—after mouse transplantation—leads to a myeloproliferative disease that is not associated with eosinophilia [70]. Moreover, human CD34+ cells lentivirally transduced with the chimeric protein displayed increased proliferation and showed an eosinophil differentiation when stimulated by eosinopoietic cytokines in a NF-kB-dependent manner [59,71].

**Figure 3:**
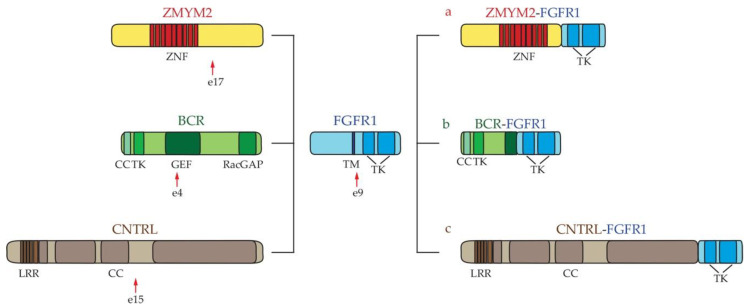
**Schematic representation of FGFR1 fusion rearrangements.** (a) *ZMYM2-FGFR1*; (b) *BCR-FGFR1*; (c) *CNTRL-FGFR1.* The arrows indicate the position of breakpoints of FGFR1. BCR = breakpoint cluster region; CC = Coiled–Coil; CNTRL = Centriolin; FGFR1 = fibroblast growth factor receptor 1; GEF = Guanine Nucleotide Exchange Factor; RacGAP = COOH-terminal GTPase Activating Protein (GAP) domain; TK = Tyrosine Kinase; LRR = Leucine-Rich Repeat; ZNF = Zing Finger; ZMYM2 = Zinc finger MYM-type protein 2.

*FGFR1 fusions*: the fibroblast growth factor receptor 1 (FGFR1) is a monomeric protein that dimerizes upon ligand binding. Dimerization drives TK activity, which promotes cell proliferation [72]. *FGFR1* abnormalities involve at least 14 gene partners generating gene fusions with different incidences. *FGFR1* rearrangements are not cryptic; hence, they can be diagnosed by conventional cytogenetic analyses [73]. The most common fusions are represented by *Zinc finger MYM-type protein 2* (*ZMYM2)-FGFR1* (*ZMYM2* exon 17–*FGFR1* exon 9: 40%), *BCR-FGFR1* (*BCR* exon 4–*FGFR1* exon 9: 18%), and *Centriolin-FGFR1* [*CNTRL (CEP110)* exon 15–*FGFR1* exon 9: 15%) [30,35,74,75] (Figure 3). In all of these pathologic fusions, the improper constitutive activation of the FGFR1 catalytic domain drives disease initiation and progression. In turn, the FGFR1 kinase phosphorylates specific tyrosine residues, promoting proliferation and pro-survival mediators, such as RAS/MAPK, PI3K/AKT, and STATs [24,72]. Hematological neoplasias that are associated with eosinophilia and expressing these chimeric proteins are MPN, ALL, and AML [24].

**Figure 4:**
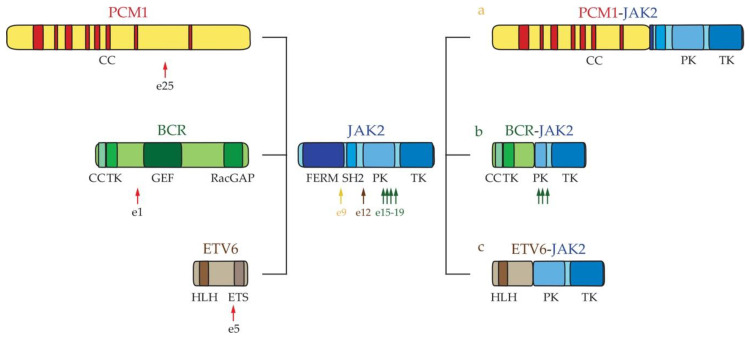
**Schematic representation of *JAK2* fusion rearrangements.** (a) *PCM1-JAK2*; (b) *BCR-JAK2*; and, (c) *ETV6-JAK2*. The arrows indicate the position of breakpoints of *JAK2.* BCR = breakpoint cluster region; CC = Coiled Coil; GEF = Guanine Nucleotide Exchange Factor; ETS = (Erythroblast Transformation Specific) DNA-binding domain; HLH = Helix-Loop-Helix oligomerization domain; FERM = 4.1 ezrin, radixin and moesin domain; JAK2 = janus kinase 2; PCM1 = Pericentriolar Material 1; PK = Pseudo kinase domain; RacGAP = COOH-terminal GTPase Activating Protein (GAP) domain; SH2 = Src-homology-2 domain; TK = Tyrosine Kinase.

*JAK2 fusions*: JAK2 is a component of the intracellular JAK/STAT pathway. The signals that are mediated by this signaling axis play a critical role in modulating the immune system through multiple cytokine receptors [76,77,78,79]. Chromosome abnormalities that are located in 9p24 involve *JAK2* and different gene partners (Figure 4) [80]. The *PCM1-JAK2* fusion (PCM1 exon 25-*JAK2* exon 9) (Figure 4a) [57] may be expressed in MPN, MDS B-ALL, or AML, and has been associated with different levels of eosinophilia in both blood and BM [57,58,81]. Additional patients have been described exhibiting rare *JAK2* rearrangements that are identified as *BCR-JAK2* (*BCR* exon 1-*JAK2* exons 15, 17, 18 or 19) (Figure 4b) and *ETV6-JAK2* (*ETV6* exon 5-*JAK2* exon 12) (Figure 4c) [82,83,84,85,86,87,88].

*PCM1*, *BCR,* and *ETV6* encode for proteins containing a coiled-coil region that mediates an oligomerization process of the ensuing chimeric proteins. This event causes constitutive activation of the JAK2 kinase increasing cell proliferation, survival, and differentiation by STAT signaling [89,90,91]. The JAK2-rearranged eosinophilia displays an unfavorable clinical course with a rapid progression from chronic to acute leukemia [85].

*Other gene fusions*: eosinophilia-associated neoplasm can be characterized by rare rearrangements involving *FLT3* and *ABL1*. The most common include *ETV6-FLT3* and *ETV6-ABL1,* which are typically identified in chronic myeloid diseases, such as eosinophilic leukemia and/or in T-ALL leukemia/lymphoma [92,93]. For both rearrangements, the *ETV6* coiled-coil region triggers an oligomerization process, which causes constitutive activation of the catalytic domains of either *FLT3* or *ABL1*. This induces the activation of the RAS/MAPK pathway, which promotes cell survival and proliferation [24].

*T-cell receptor gene rearrangements*: IL-5 overproduction by activated mature T-cells, leading to the polyclonal expansion of eosinophils, has been reported in several studies [23,32,94,95]. Therefore, the detection of an aberrant T-cell immunophenotype by clonal TCR gene rearrangement is required for the diagnosis of most L-HES patients [95,96]. T-cell clonality is not detected in all patients with demonstrated aberrant lymphocyte cells [36]. However, a negative analysis may not reflect the true absence of clonality, as the clonal nature of the disease may go undetected, due to a lack of sensibility when the aberrant cells represent a small proportion of the total lymphocyte population. In these patients, Roufosse and colleagues suggest repeating clonality testing after the purification of aberrant T-cells and analyzing T-cell cytokine secretion profiles by measuring the concentrations in supernatants of cultured peripheral blood mononuclear cells [36]. It should be noted that a high portion of HES_US_ patients (18/42 patients, 43%) exhibit a clonal TCR gene rearrangement by PCR, although it is unclear whether such clonal T-cell population is always the cause of the disease [96].

### Next Generations Sequencing Approaches to Investigate DNA Mutations in Patients with Eosinophilic Disorders

In the recent years, the next generation sequencing (NGS) [77] has been used in the identification of numerous mutations in a large proportion of myeloproliferative disorders and/or AML and CML patients [97,98,99,100,101]. Moreover, different studies investigated the use of NGS-based mutation panels to study HE patients. Baer and colleagues reported that somatic mutations are more frequent in patients with *FGFR1* rearrangements when compared to those with *PDGFRα*, *PDGFRβ*, or *PCM1-JAK2* alterations. For example, 83% of *FGFR1*-rearranged individuals harbored *RUNX1* mutations [102]. Two additional studies reported a wide range of mutation frequencies (11–28%) in different cohorts of 98 and 51 patients with HEus and/or HESus [103,104]. By performing an NGS panel that was designed for myeloid neoplasias, Wand et al. found that, in 51 idiopathic HES individuals, the most frequently mutated genes are ASXL t*ranscriptional regulator 1* (*ASXL1)* (43%), *Tet methylcytosine dioxygenase 2* (*TET2)* (36%), *Enhancer of zeste homolog 2(EZH2)* (29%), *SET binding protein 1 (SETBP1)* (22%), *Casitas B-lineage Lymphoma* (*CBL)* (14%), and *Notch homolog 1, translocation-associated* (*NOTCH1)* (14%) [104]. The authors provided evidence of clonality for subjects with clinical or morphologic features that are suggestive of neoplasia and/or contributed to the diagnosis of CEL, NOS [104]. A Korean study investigated T-cell clonality and the impact of the mutations in 30 individuals that were diagnosed with HEus/HESus by performing NGS, TCR gene rearrangement assays, and a pathway network analysis [105]. They found that a 53.3% mutation frequency with the most frequently altered genes *NOTCH1* (26.7%), *Scribble Planar Cell Polarity Protein* (*SCRIB)*, and *Stromal Antigen 2* (*STAG2)* (16.7%) and *SH2B adapter protein 3* (*SH2B3)* (13.3%). They also identified 5 (*MAPK1*, *RUNX1*, *GATA2*, *NOTCH1*, and *TP53*) out of 21 candidate genes functionally linked to the eosinophilopoietic pathways and observed that 13.3% of patients had a clonal TCR rearrangement. The study suggested that mutations affecting eosinophilopoiesis highlighted a special subgroup of IHE/IHES and that these mutations were more likely to be associated with a clonal eosinophil proliferation [105]. Finally, Cross and colleagues found an activating *STAT5B* N642H driver mutation in 1.6% of patients with lymphoproliferative disorders that were referred for eosinophilia. The authors demonstrated that individuals with additional mutated genes, other than *Splicing factor 3B subunit 1* (*SF3B1),* had an inferior overall survival (OS) when compared to those with the *STAT5B* mutation alone [106].

## 4. Therapeutic Option for Patients with Eosinophilic Disorders

**Figure 5:**
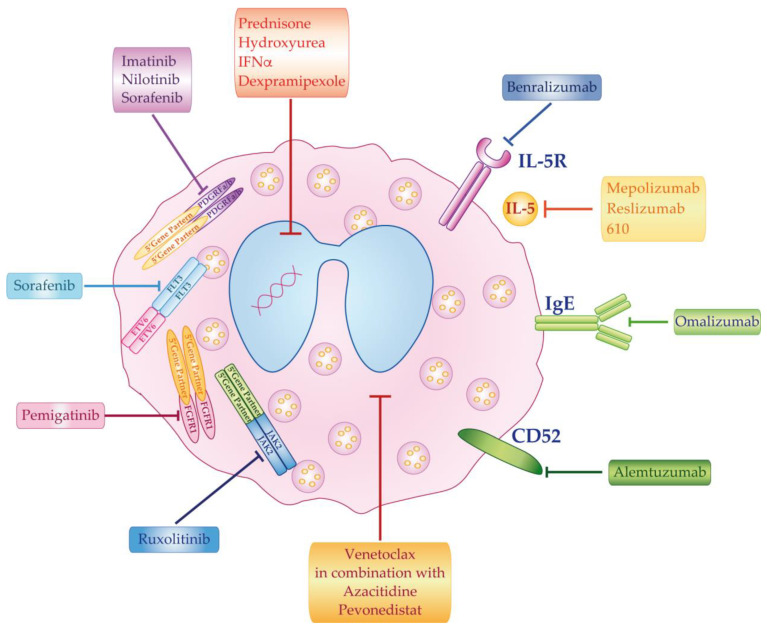
**Working model depicting the mechanism of action of pharmacological agents used in hypereosinophilia disorders.***Alemtuzumab* = anti-CD52 monoclonal antibody; *Benralizumab* = anti-IL5-R monoclonal antibody; *Dexpramipexole*, *Hydroxyurea* and *IFNα* = cytotoxic agents*; Imatinib* and *Nilotinib* = PDGFRs inhibitors; *Pegatinib* = FGFR1 inhibitor; *Mepolizumab*, *Reslizumab* and *610* = recombinant anti-IL5 humanized monoclonal antibody; *Omalizumab* = anti-IgE monoclonal antibody; *Prednisolone* = corticosteroid; *Ruxolitinib* = Jak2 inhibitor; *Sorafenib* = PDGFRα and FLT3 inhibitor; *Venetoclax* in combination with *Azacitidine* and *Pevonedistat* = chemotherapy regimens.

The best clinical treatment of HES depends on disease etiology and subtypes. However, even in the absence of a known cause, HES must be promptly treated in order to reduce potential morbidity that can result from organ damage. In this regard, an AEC of 350–500/mm^3^ has been recommended as a threshold for starting treatment [23]. Multiple therapeutic approaches are currently employed (Table 2) with further compounds under investigation or in ongoing trials (Table 3). The main therapeutic options for HES patients can be divided in five groups: corticosteroids, cytotoxic agents, tyrosine kinase inhibitors, monoclonal antibodies (mAb), and chemotherapy (Figure 5, Table 2 and Table 3).

### 4.1. Corticosteroids

This group of drugs is the current mainstay for slowing and/or preventing organ damage and it can be used as first-line therapy in patients with strictly defined HES. Because steroid therapy can be complicated by side effects in patients requiring long-term treatment, different studies have been conducted in order to better define the right steroid dose [107,108]. An ongoing study (NCT 01524536) is trying to determine whether a single *prednisone* dose can be used to predict which subjects with hypereosinophilia will respond to treatment with individuals developing symptom recurrence or an increase in their AEC requiring the addition of a second drug, such as hydroxyurea (HU) (Table 3).

### 4.2. Cytotoxic Agents

The main drugs that are employed for the treatment of HES are *hydroxyurea* (as a first-line agent or in combination with corticosteroids in non-responders patients) [29,109] and *Interferona* (IFNα), with the latter drug used as a second-line agent after steroid-failure (Figure 5 and Table 2). *IFNα* can also be used in conjunction with corticosteroids or as a steroid-sparing agent for patients requiring higher doses of prednisone or presenting contraindications to steroid therapy [110,111,112]. In addition to *HU* and *IFNα*, *dexpramipexole* is an orally bioavailable synthetic aminobenzothiazole, which, in a non-randomized, proof-of-principle study, reduced blood and tissue eosinophils and enabled corticosteroid reduction or cessation in HES patients (Figure 5 and Table 3) [113]. Currently, a trial (NCT02101138) is evaluating whether dexpramipexole can reduce the steroid dose that is needed to control eosinophilia and HES symptoms (Table 3).

### 4.3. Tyrosine Kinase Inhibitors

*Imatinib and Nilotinib:* currently used as ABL1-directed inhibitors for CML patients [114,115,116], they were considered to be possible therapeutic agents in HES for their ability to inhibit PDGFR kinase activity. However, to date, only imatinib has been approved as a first-line treatment for patients with myeloid disease, with eosinophilia expressing *FIP1L1-PDGFRα* or carrying other *PDGFRα* or *PDGFRβ* fusions [107,117] (Table 2). The Food and Drug Administration (FDA)-recommended starting dose for patients with the *FIP1L1-PDGFRα* rearrangement is 100 mg daily, which is sufficient to achieve complete hematologic and molecular remissions. For patients with myeloid neoplasms and eosinophilia expressing *PDGFRβ* fusions, the recommended starting dose is 400 mg, being lowered to 100 mg during maintenance [117]. A phase 2-trial is evaluating the safety and efficacy of a combination of imatinib and ruxolitinib in reducing peripheral blood eosinophilia in patients with the myeloid form of HES (NCT00044304) (Table 3 and Figure 5). Furthermore, the experimental data obtained while using rat or mouse models demonstrated that ABL inhibition by imatinib reduces the TGF-B1 profibrogenic activity in renal and lung tissues interested from eosinophil disorders [118,119].

An ongoing study designed as a managed access program presently allows access to nilotinib for eligible patients that were diagnosed with HES (NCT04498871) (Table 3 and Figure 5).

*Pemigatinib:* INCB054828 is an oral *FGFR1*, *2* and *3* inhibitor that is currently under evaluation in *FGFR1*-rearranged myeloid/lymphoid neoplasms [120].

*Ruxolitinib* and *Sorafenib:* these two multi-kinase inhibitors should be considered to be a bridge to HSCT for patients displaying the *JAK2* or *FLT3* tyrosine kinase fusions, respectively (Table 2 and Figure 5) [92,121,122,123]. Ruxolitinib is also under investigation in HES patients expressing the *BCR-JAK2* fusion, in order to determine their overall hematologic response to this drug (NCT03801434) (Table 3). In addition, Lierman and colleagues reported that Sorafenib seems to be an in vitro potent inhibitor in *FIP1L1-PDGFRα* rearranged patients with T674I mutation [124].

*Monoclonal antibodies:* several FDA-approved antibodies have shown benefit in reducing circulating eosinophils, either by targeting eosinophilopoietic cytokines, or by depleting eosinophils via antibody-dependent cellular cytotoxicity. The antibodies that are presently available or undergoing clinical trials are directed against IL-5, the IL-5 receptor, IgE, or the CD52 antigen.

*Mepolizumab:* a fully humanized monoclonal IgG antibody that inhibits the binding of IL-5 to the chain of the IL-5 receptor expressed on eosinophils, reducing their survival and TGF-B production [125,126,127]. The FDA has currently approved this compound for severe asthma and eosinophilic granulomatosis with polyangiitis, but not for HES. It is available on a compassionate use-bases for individuals with life-threatening HES who have failed at least three standard lines of treatment (NCT00244686) (Table 3 and Figure 5).

*Reslizumab*: a humanized anti-IL5 IgG4 monoclonal antibody approved by the FDA for severe eosinophilic asthma that has not yet been studied in HES [128] (Table 3 and Figure 5).

*Benralizumab:* an anti-IL5 receptor antibody that is employed in patients with severe, uncontrolled asthma [129], with initially unsatisfactory results in HES [130]. After binding to IL-5R, eosinophils become a target for destruction by NK cells via antibody-dependent cell-mediated cytotoxicity [131]. Currently, a phase 3 study is evaluating the efficacy and safety of benralizumab for HES patients (NCT04191304). A second trial (NCT02130882) is testing the ability of this drug to safely decrease eosinophils in individuals that are diagnosed with HES (Table 3 and Figure 5).

*Omalizumab*: an anti-IgE monoclonal antibody approved by the FDA for the treatment of asthma and chronic spontaneous urticaria. It showed promising activity in some eosinophilic disorders, although results were not as consistent as those that were seen with anti-IL-5 or anti-IL-5R antibodies (Table 2 and Figure 5) [132]. The drug may lead to the inhibition of the release of cytochines, such as IL-4, IL-5, and IL-13, as these are responsible for eosinophils recruitment and activation [132].

*Alemtuzumab*: an anti-CD52 monoclonal antibody that has been evaluated in HES_us_ based on the expression of the CD52 antigen on the eosinophil surface (Table 2 and Figure 5) [133,134]. Verstovsek and colleagues found that alemtuzumab achieved a complete hematologic remission in 10/12 (83%) patients with refractory HES and a partial remission in the remaining two subjects [135].

Finally, chemotherapy, as well as autologous stem cell transplant, are usually employed for patients with eosinophilic leukemia, T-cell lymphomas, or other types of primary HES that are refractory to alternative treatments [136,137]. As an alternative to chemotherapy, an ongoing phase I/II trial (NCT03862157) is studying the association of *venetoclax*, *azacitidine*, and *pevonedistat* in patients with newly diagnosed acute myeloid leukemia and other hematological disorders, including CEL-NOS (Table 3 and Figure 5).

## 5. Conclusions

Eosinophilic disorders represent a group of highly heterogeneous diseases that are characterized by various degrees of persistent blood and/or tissue hypereosinophilia with potential for end-organ dysfunction [2,23]. Hence, a timely diagnosis is essential and it requires a combination of histopathologic, immunophenotypic, cytogenic, and molecular analyses. The identification of specific and recurrent genetic alterations in HES suggests the possible use of molecularly targeted therapies that have proven to be successful in many tumor types, as this approach selectively kills neoplastic cells that harbor a specific molecular hallmark [138,139,140,141,142,143,144].

In this context, the distinction of each HES variant is critical for the appropriate management of the disease. The development of non-invasive sampling methods, coupled with an extensive NGS-based molecular characterization, will be important in distinguishing the different disease variants and discriminating an eosinophil myeloid neoplasia from HE_US_. Moreover, this approach will enable an accurate disease monitoring, promptly identifying patients with a rapidly progressing hematological malignancy.

The recent focus of HES is based on the increasing of availability compounds targeting different mediators and the cells involved in the mechanism of the disease.

Different ongoing clinical trials (Table 3) based on different drugs, used alone or in combination, will allow for a better understanding of the best initial therapy for any single patients with HES while taking the individual pathogenesis into consideration. Indeed, more targeted approach to treatment need an implementation of significative changes in the way that patients are managed through a more personalized approach to prognostication, the prediction of treatment responses.

Finally, with increasing use of anti-IL-5 or anti-IL-5R antibodies for hypereosinophilic disease in clinical practice, in the near future the focus should be on optimizing doses and regimens. A better combination of different active molecules will be investigated in order to design efficacious and minimally toxic tailored treatment regiments for patients with these complex disorders.

## Figures and Tables

**Table 1:** Molecular pathogenesis in Hypereosinophilic Syndrome.

**Fusion Genes**			
**Gene**	**Translocation**	**Gene**	**Translocation**
***PDGFR*** *α*		***PDGFR*** *β*	
*FIP1L1-PDGFR* *α*	del(4)(q12;q12)	*ETV6-PDGFR* *β*	t(5;12)(q33;p13)
*BCR-PDGFR* *α*	t(4;22)(q12;q11)		
***FGFR1***		***JAK2***	
*ZMYM2-FGFR1*	t(8;13)(p11.2;q12.1)	*PCM1-JAK2*	t(8;9)(p22;p24)
*CNTRL-FGFR1*	t(8;9)(p11.2;q33.2)	*BCR-JAK2*	t(9;22)(p24;q11.2)
*BCR-FGFR1*	t(8;22)(p11.2;q11.2)	*ETV6-JAK2*	t(9;12)(p24;p13)
**Other Genes**			
*ETV6-FLT3*	t(12;13)(p13;q12)		
*ETV6-ABL1*	t(9;12)(q34;p13)		
**Receptor Rearrangements**			
*T Cell receptor rearrangement*			
**Mutated Genes**			
**Genes**	**Percentage of mutation**	**Genes**	**Percentage of mutation**
*RUNX1*	83%	*SETBP1*	22%
*ASXL1*	43%	*CBL*	14%
*TET2*	36%	*NOTCH1*	14%
*EZH2*	29%		

**Table 2:** Therapeutic options for eosinophilic disorder.

Drug	Mechanism of Action	Dose	Target Neoplasm
**Corticosteroids**			
Prednisone	Slow and prevent end-organ damage	1 mg/kg daily	HES
**Cytotoxic agents**			
Hydroxyurea	Inhibit DNA synthesis	500–1000 mg/daily	HES (+ corticosteroids); Steroid non-responders.
IFNα	Inhibit cell growth and induct apoptosis	Initiation: 1 million units tiw * Escalation: 3–4 million units tiw *	HES (+ corticosteroids); HES & CEL, NOS refractory to other therapies; Lymphocyte-variant hypereosinophilia.
**Targeted therapies**			
**TKIs**			
Imatinib	Inhibit both TGFb and PDGF-R pathway	100–400 mg/daily	*PDGFRα* rearranged; *PDGRFβ* rearranged; Alternate *PDGRFβ* fusions; Selected cases HES and CEL, NOS.
Ruxolitinib	Inhibit dysregulated JAK/STAT signalling pathway	20 mg PO BID	Eosinophilic leukemia with the *PCM1-JAK2* fusion [t(8;9)(p22;p24)].
Sorafenib	Inhibit several kinases involved in both tumour cell proliferation and angiogenesis	400 mg/twice daily	*FIP1L1-PDGFRα* rearranged pts with T674I mutation; FLT3-rearranged cases.
**Monoclonal antibodies**			
**Anti-IL-5**			
Mepolizumab	Inhibit binding of IL-5 to the α chain of the IL-5R	100–300 mg every 4 weeks	Eosinophilic asthma and eosinophilic granulomatosis with polyangiitis.
Reslizumab	Inhibit the proliferation of eosinophils by binding to the α chain of the IL-5R	1 mg/kg	Eosinophilic asthma and eosinophilic esophagitis.
**Anti-IL-5R**			
Benralizumab	Inhibit hetero-oligomerization of α and β subunits of IL-5R	30 mg by subcutaneous injection every 4 weeks	Severe asthma.
**Anti-IgE**			
Omalizumab	Inhibit release of cytokines such as IL-4, IL-5, and IL-13; block unbound IgE.	dose/frequency calculated bases on weight per serum IgE	Eosinophilic disorders, in particular asthma/nasal polyps
**Anti-CD52**			
Alemtuzumab	Mediate the lysis of CD52+ cells	5–30 mg 1 to 3 times weekly	Refractory HES pts.

* subcutaneous injection three times a week. IFNα: Interferon alfa; HES: Hypereosinophilic Syndrome; CEL, NOS: Chronic Eosinophilic Leukemia, Not Otherwise Specified; pts: patients; IL5: Interleukin5; IL-5R: Interleukin5 Receptor; PO BID: Orally twice day; PDGF-R: Platelet-Derived Growth Factor Receptor; TGFb: Transforming Growth Factor-b; TKIs: Tyrosine kinases inhibitors.

**Table 3:** Ongoing clinical trials for eosinophilic disorder.

Drug	Combination	Target	Design	Patients	Identifier	Phase	Status
**Corticosteroids**							
Prednisone	-	-	Single Group Assignment; Open Label	100	NCT01524536	Phase IV	Recruiting
Dexpramipexole	-	-	Non-Randomized; Single Group Assignment; Open Label	15	NCT02101138	Phase II	Unknown
**Targeted therapies**							
**TKIs**							
Imatinib	Ruxolitinib	*FIP1L1-PDGFRα* & *PDGFRβ*	Non-Randomized; Sequential Assignment; Open Label	60	NCT00044304	Phase II	Recruiting
Nilotinib	-	*FIP1L1-PDGFRα* & *PDGFRβ*	*NP*	*NP*	NCT04498871	*NP*	Available
Ruxolitinib	-	*BCR-JAK2* Fusion Protein Expression	Single Group Assignment; Open Label	25	NCT03801434	Phase II	Not yet recruiting
**Monoclonal antibodies**							
Mepolizumab		IL-5	*NP*	*NP*	NCT00244686	*NP*	Available
610 *	Placebo	IL-5	Randomized; Parallel Assignment	52	NCT04445038	Phase I	Recruiting
Benralizumab	-	IL-5R	Multicentre; randomised; double-blind; parallel Assignment	120	NCT04191304	Phase III	Not yet recruiting
	Placebo		Randomized; Parallel Assignment	22	NCT02130882	Phase II/III	Active, not recruiting
**Chemotherapy**							
Venetoclax	Azacitidine, Pevonedistat	-	Single Group Assignment; Open Label	40	NCT03862157	Phase I/II	Recruiting

IL5: Interleukin5; IL-5R: Interleukin5 Receptor; NP: Not provided. * Recombinant anti-IL5 humanized monoclonal antibodies.

## Abbreviations

ABL1	Abelson murine leukemia
AEC	Absolute Eosinophil Count
ALL	Acute Lymphoblastic Leukemia
BCR	Breakpoint cluster region
CEL-NOS	Chronic Eosinophilic Leukemia non otherwise specified
CML	Chronic Myeloid Leukemia
CMML	Chronic Myelomonocytic Leukemia
CNL	Chronic Neutrophilic Leukemia
*CNTRL*	Centriolin
*FGFR1*	Fibroblast growth factor receptor 1
FIPL1	FIP1-like 1
FLT3	fms-like tyrosine kinase 3
GM-CSF	Granulocyte-Macrophage Colony-Stimulating Factor
GUSβ	β-glucuronidase
HE	Hypereosinophilia
HES	Hypereosinophilia syndrome
IL-5	Interleukin 5
JAK2	janus kinase 2
MDS	myelodysplastic sindromes
MPN	myeloproliferative neoplasms
PDGFRα	Platelet-Derived Growth Factor Receptor alpha
PCM1	Periocentriolar material 1
TARC	Thymus and activation-regulated chemokine
TK	Tyrosine kinase
TKI	Tyrosine kinase inhibitor
TFR	Treatment-free remission
ZMYM2	Zinc finger MYM-type protein 2
